# Ancient Evolutionary Trade-Offs between Yeast Ploidy States

**DOI:** 10.1371/journal.pgen.1003388

**Published:** 2013-03-21

**Authors:** Enikö Zörgö, Karolina Chwialkowska, Arne B. Gjuvsland, Elena Garré, Per Sunnerhagen, Gianni Liti, Anders Blomberg, Stig W. Omholt, Jonas Warringer

**Affiliations:** 1 Centre for Integrative Genetics (CIGENE), Department of Animal and Aquacultural Sciences, Norwegian University of Life Sciences (UMB), Ås, Norway; 2 Department of Chemistry and Molecular Biology, University of Gothenburg, Gothenburg, Sweden; 3 Centre for Integrative Genetics (CIGENE), Department of Mathematical Sciences and Technology, Norwegian University of Life Sciences (UMB), Ås, Norway; 4 IRCAN, CNRS UMR 6267, INSERM U998, University of Nice, Nice, France; 5 NTNU Norwegian University of Science and Technology, Faculty of Natural Sciences and Technology, Department of Biotechnology, Trondheim, Norway; University of British Columbia, Canada

## Abstract

The number of chromosome sets contained within the nucleus of eukaryotic organisms is a fundamental yet evolutionarily poorly characterized genetic variable of life. Here, we mapped the impact of ploidy on the mitotic fitness of baker's yeast and its never domesticated relative *Saccharomyces paradoxus* across wide swaths of their natural genotypic and phenotypic space. Surprisingly, environment-specific influences of ploidy on reproduction were found to be the rule rather than the exception. These ploidy–environment interactions were well conserved across the 2 billion generations separating the two species, suggesting that they are the products of strong selection. Previous hypotheses of generalizable advantages of haploidy or diploidy in ecological contexts imposing nutrient restriction, toxin exposure, and elevated mutational loads were rejected in favor of more fine-grained models of the interplay between ecology and ploidy. On a molecular level, cell size and mating type locus composition had equal, but limited, explanatory power, each explaining 12.5%–17% of ploidy–environment interactions. The mechanism of the cell size–based superior reproductive efficiency of haploids during Li^+^ exposure was traced to the Li^+^ exporter *ENA*. Removal of the Ena transporters, forcing dependence on the Nha1 extrusion system, completely altered the effects of ploidy on Li^+^ tolerance and evoked a strong diploid superiority, demonstrating how genetic variation at a single locus can completely reverse the relative merits of haploidy and diploidy. Taken together, our findings unmasked a dynamic interplay between ploidy and ecology that was of unpredicted evolutionary importance and had multiple molecular roots.

## Author Summary

Organisms vary in the number of chromosome sets contained within the nucleus of each cell, but neither the reasons nor the consequences of this variation are well understood. We designed yeasts that differed in the number of chromosome sets but were otherwise identical and mapped the consequences of such ploidy variations during exposure to a large palette of environments. Contrary to commonly held assumptions, we found ploidy effects on the mitotic reproductive capacity of yeast to be the rule rather than the exception and to be highly evolutionarily conserved. Furthermore, our data rejected previously contemplated hypotheses of generalizable advantages of haploidy or diploidy when cells face nutrient starvation or are exposed to toxins or increased mutation rates. We also mapped the molecular processes mediating ploidy–environment interactions, showing that cell size and mating type locus composition had equal explanatory power. Finally we show that ploidy effects can be mechanistically very subtle, as a designed shift from one plasma membrane Li^+^ transporter to another completely altered the relative merits of having one or two chromosome sets when exposed to high Li^+^ concentrations. This complex and dynamic interplay between the number of chromosomes sets and the fluctuating environment must be taken into account when considering organismal form and behavior.

## Introduction

A central yet poorly understood variable of life is the number of chromosome sets contained within the nucleus of eukaryotic cells. Ploidy varies throughout the tree of life, with ancient polyploidization events close to the angiosperm [1], [2] and vertebrate [3], [4] radiations and among yeasts [5]. Variation in ploidy states was initially predicted to be neutral as the balance between genes was assumed to be unperturbed [6]. However, it has recently become clear that ploidy has substantial impacts, defining genome evolution and heredity [7], controlling organismal development through transient establishment of specialized polyploid cell types [8] and promoting tumor progression [9]. Despite the biological impact of ploidy differences, the underlying molecular, evolutionary and ecological constraints controlling these remain murky [10]. Mutational models are based on chromosome set additions increasing the number of mutable sites but masking recessive variation, thereby affecting the emergence, tolerance to and purging of *de novo* mutations [11]. Factors such as strength of selection, mutation rate, population size and ratios of deleterious to adaptive and recessive to dominant mutations consequently determine whether a particular ecological context will favor high or low ploidy [10], [12]–[14]. In contrast, cell size models presuppose higher ploidy states to increase cell and organelle volume but to fail to proportionally enlarge surface areas [15], thereby distorting the balance between transport rates, costs and needs. In these models, the abundance of beneficial and harmful substances imposes selection for different ploidy states in different environments [15]–[18]. Finally, life history models note the intricate interlacing of ploidy variation with alterations of mating, meiosis and sporulation patterns, which originate in the ploidy dependent genetic composition at sex determining loci and the resulting ploidy dependent initiation of dedicated transcriptional programs [19]. This potentiates co-selection on ploidy and ability to mate, outbreed and sporulate in response to mostly unknown environmental cues.

The unicellular baker's yeast, *Saccharomyces cerevisiae*, reproduces asexually in stable haploid, diploid and polyploid forms and has emerged as a key model for ploidy research. Here, we exhaustively mapped the impact of ploidy on the mitotic fitness of *S. cerevisiae* and its never domesticated relative *Saccharomyces paradoxus* across wide swaths of their genotypic and phenotypic space. Influences of ploidy on asexual proliferation in different ecological contexts were found to be the rule rather than the exception with the majority of ploidy effects being well conserved over the 2 billion generations separating the two species [20]. This demonstrates preservation in the face of considerable genetic drift and large ecological upheavals. Previous hypotheses of generalizable advantages of haploidy or diploidy in ecological contexts imposing nutrient restriction, toxin exposure and elevated mutational loads were rejected in favor of more fine-grained models of the interplay between ecology and ploidy. Cell size and mating type locus composition each explained 12.5–17% of ploidy effects in the universal reference strain S288c.

## Results

### No overall asexual reproductive advantage of haploidy or diploidy

**Figure 1:**
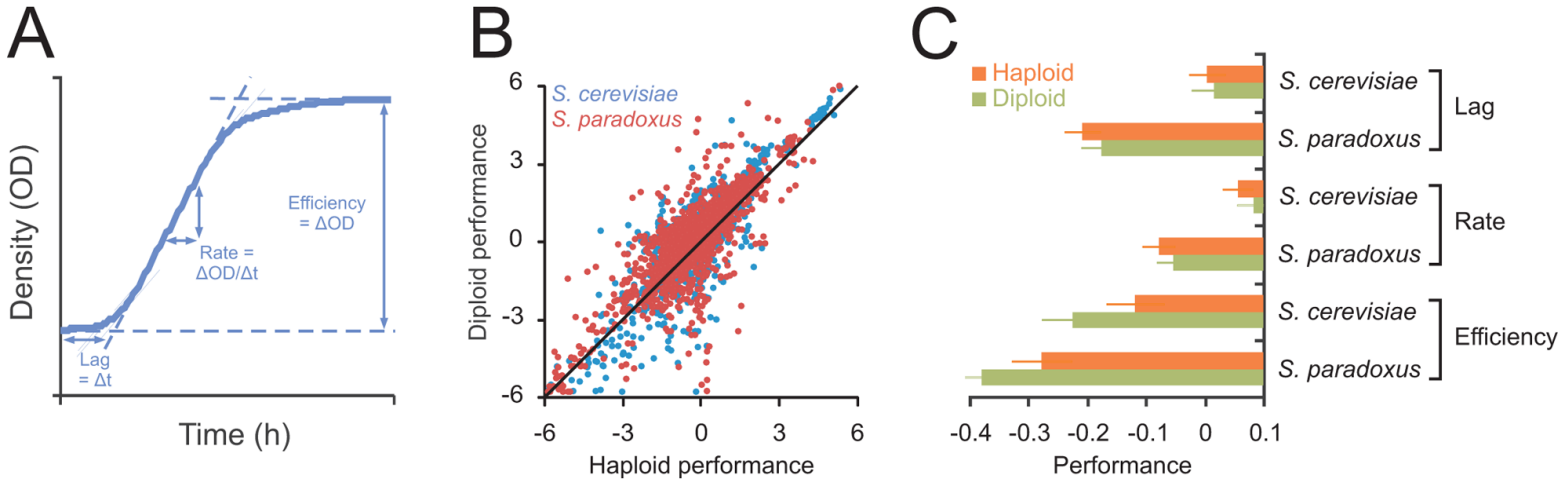
Ploidy–environment interactions are the rule rather than the exception in yeast and favor haploidy and diploidy equally. A) The mitotic fitness components lag (time to initiate proliferation), rate (population doubling time) and efficiency (total change in population density) of asexual reproduction were extracted from high density growth curves of 24 *S. cerevisiae* and 27 *S. paradoxus* strains cultivated as haploids (n = 4) and diploids (n = 2) in an array of environmental contexts. Performance was log(2) transformed and normalized to that of the universal reference strain S288c, providing relative performance measures. B) The performances of haploids and diploids were compared over all species, strains, mitotic fitness components and environments. Line indicates the 1∶1 correlation. C) The performance of haploids and diploids over all strains and environments. Note that performance is on a log(2) scale. No significant difference between the two ploidy states (FDR, α = 0.05) were found. Error bars represent SEM.

To map the impact of ploidy on the capacity for asexual reproduction across the genomic and phenotypic space of the species, 24 *S. cerevisiae* and 27 *S. paradoxus* natural isolates (Table S1) were propagated clonally as haploids and *MAT* ***a***/α autodiploids in 33 distinct environments (Table S2). Together, these isolates represented >90% of the known genetic [21] and phenotypic [22] variation within these species and encompassed the major populations, geographic origins and source environments (Table S1). From >12.000 high density population growth curves, we extracted the mitotic fitness components lag (population adaption time), rate (population doubling time) and efficiency (population density change) of clonal reproduction (Figure 1A). These measures together encapsulate the capacity of yeast for asexual proliferation, the dominant mode of yeast reproduction in the wild [23], [24], and are thus likely to influence yeast fitness substantially in natural contexts. Considering the complete range of environmental and genetic contexts, the performance of haploids and diploids adhered closely to the 1∶1 null hypothesis expectation of overall equal performance of haploids and diploids (Figure 1B). The tendency towards similar performance of haploids and diploids was evident for all mitotic fitness components, for both species and for all populations, source habitats and genetic backgrounds (Figure 1C, Figures S1 and S2). Hence, considering a wide section of environmental space, we conclude that evolution has failed to establish a decisive asexual reproductive advantage of either haploidy or diploidy.

### Ploidy–environment interactions are the rule rather than the exception and of ancient evolutionary origin

**Figure 2:**
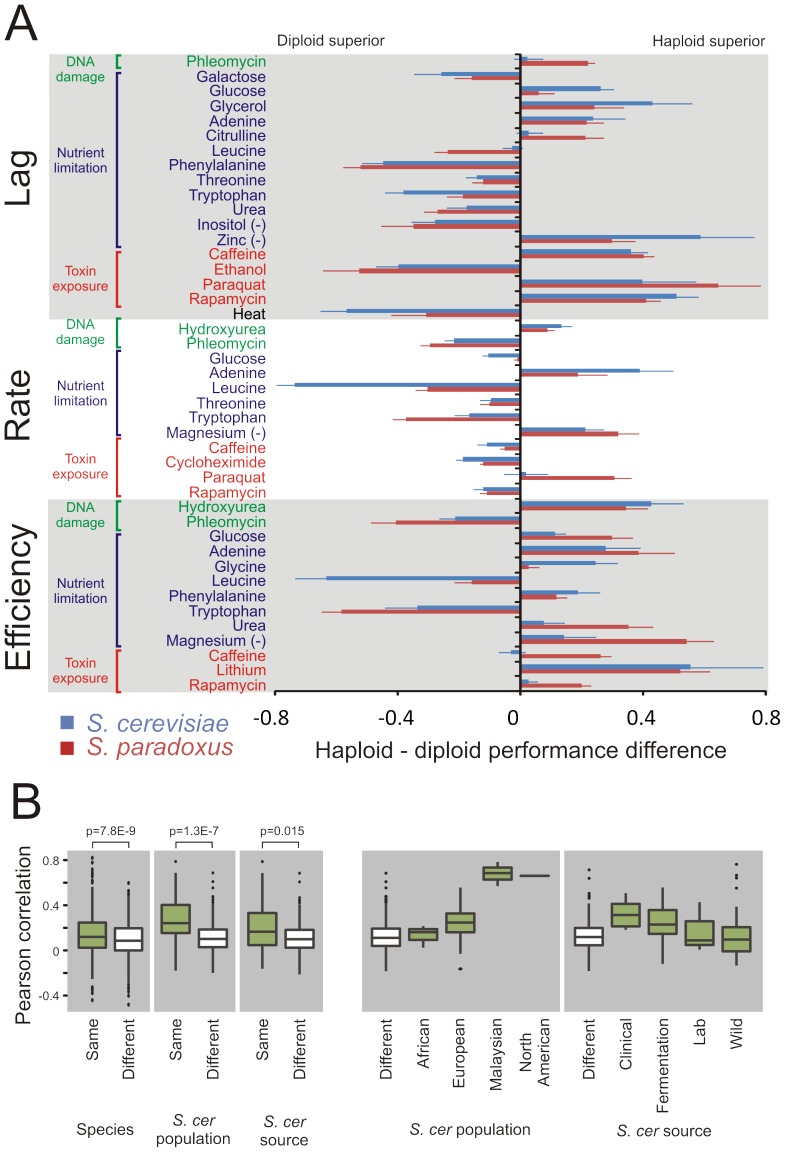
Ploidy–environments interactions are conserved since before the *S. cerevisae* and *S. paradoxus* radiation. A) Fitness component measures with a significant (FDR, α = 0.05) difference in performance between haploids and diploids in *S. cerevisiae*, in *S. paradoxus* or in both species. To compare haploid and diploid asexual proliferative capacity, a mean of the log(2) relative performance of the two haploid mating types (each n = 2) was used to derive a single measure of haploid performance. This was compared to that of the diploid (n = 4), by calculating the mean difference between haploid and diploid phenotypes. Each species was treated separately. Error bars represent the SEM (n = 24 for *S. cerevisiae*, n = 27 for *S. paradoxus*). B) Left panels show pairwise Pearson correlation coefficients, based on ploidy effects over all mitotic traits, between strains belonging to the same (627 pairs) or different (648 pairs) species, the same (43 pairs) or different (233 pairs) *S. cerevisiae* population and the same (65 pairs) or different (211 pairs) *S. cerevisiae* source environment. Species, population and source environment, all have significant impact on ploidy effects (ANOVA F-test; p-values displayed, note the large sample size for the between/within species comparison, and the correspondingly low SEM), but explained only 2.5%, 9.3% and 1.8% of the overall variation in correlation coefficients (R2-adj). Right panels resolve *S. cerevisiae* populations into the Malaysian, European, African and North American populations and *S. cerevisiae* sources into Clinical, Fermentation, Lab and Wild strains. Top and bottom of boxes represent 25th and 75th quartiles, bands represent medians, whiskers show the lowest and highest data point still within 1.5 interquartile range of the lower and upper quartile respectively and filled circles represent data points outside this range.

Despite the absence of a general mitotic advantage of either haploid or diploid genome architecture, ploidy dramatically affected the mitotic capacity in distinct ecological contexts (Figure 2A). Taking the complete genomic space of the two species into account, significant (FDR, α = 0.05) differences between ploidy states were observed in a vast (70%) majority of all environments, including in optimal conditions (Figure S3). Thus, ploidy is more likely than not to affect the asexual proliferation of yeast in any given environmental context. Considering haploid and haploid strains separately, the radiation into *S. cerevisiae* and *S. paradoxus* was a key determinant of phenotype variation, explaining 25.2% (ANOVA F-test, p = 1.2E-82) and 14.5% (ANOVA F-test, p = 1.7E-45) of the variance in strain pair similarity. However, the species divergence had essentially no impact on the effect of ploidy on traits, explaining only 2.5% (ANOVA F-test, p = 7.8E-9) of the similarity between strains with regards to ploidy-environment interaction (Figure 2B). In fact, the majority of significant ploidy effects were strikingly evident in both *S. cerevisiae* and *S. paradoxus* (Figure 2A). Thus, despite substantial trait differentiation during the 2 billion generations having passed since species radiation, many of the ploidy effects have remained conserved, although with substantial quantitative variations between species. To further explore the evolutionary origin of ploidy effects in *S. cerevisiae*, we estimated the degree to which the historical separation into distinct populations could explain the variation in ploidy effects, population structure being the major determinant of trait variation among *S. cerevisiae* strains [22]. However, population structure explained only 9.3% of variation in ploidy-environment interactions within *S. cerevisiae*. The North American and Malaysian populations showed virtually identical ploidy effects within populations, fully accounting for this explanatory power (Figure 2B). The later, human enforced separation of *S. cerevisiae* into clinical, fermentation, lab and wild strains only accounted for a further 1.8% of the variation in ploidy effects (Figure 2B). This is in line with the generally limited explanatory power of human influence on *S. cerevisiae* trait differentiation [22]. Taken together, our observations suggest ploidy-environment interactions to have originated in the period of shared evolutionary history of *S. cerevisiae* and *S. paradoxus*. Since the divergence of these species, these ploidy-environment interactions have resisted both natural and human imposed genetic drift and selection, consistent with the action of strong selection. The quantitative differences between the species are in line with that the strength of selection acting on each type of ploidy-environment interaction, although present in both species, has diverged somewhat during their recent, separate evolution.

### Elevated mutation rates, toxin exposure, and nutrient restriction fail to favor either ploidy state

**Figure 3:**
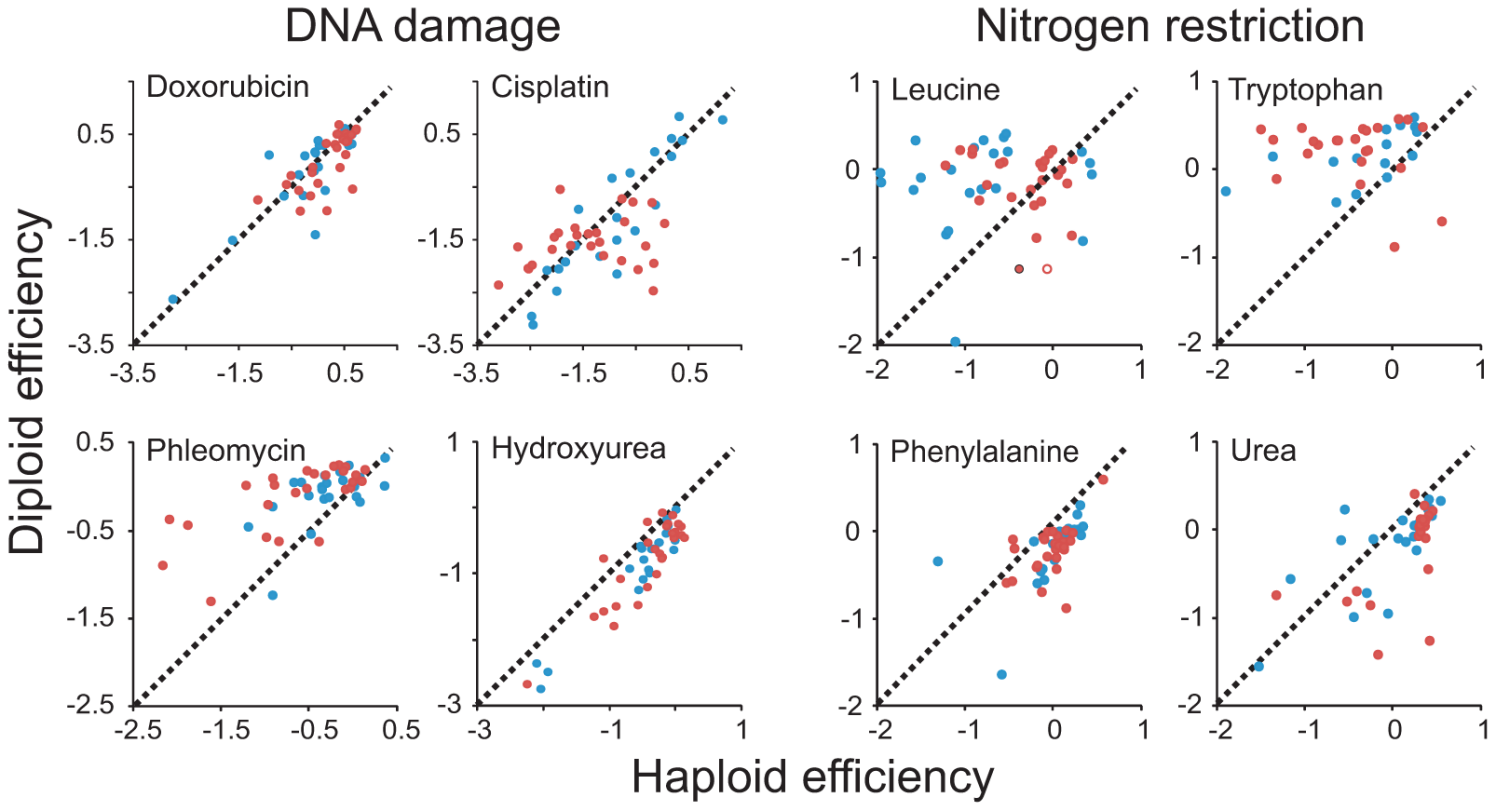
Patterns of ploidy–environment interactions refute generalizing hypotheses on the effects of mutational load, toxin exposure, and nutrient restriction. Performance of haploid (n = 4) and diploid (n = 2) versions of individual *S. cerevisiae* (blue) and *S. paradoxus* (red) strains in DNA damage inducing environments and nitrogen restricted environments. Note that data is shown on a log(2) scale. Broken lines indicate the 1∶1 correlation (null hypothesis expectation).

Environmental contexts were selected specifically to allow testing of hypotheses on the beneficial effects of diploidy in environments elevating mutational loads and in environments rich in toxic substances and of beneficial effects of haploidy during nutrient restrictions. Our data failed to support a general mitotic fitness advantage of diploidy in environments associated with elevated mutation rates. Instead, the type of DNA damage induced appeared to define the relative merits of a haploid and diploid asexual proliferation. Phleomycin, inducing DNA lesions via a free radical based mechanism, clearly favored diploidy across the genomic range of both species (Figure 2A, Figure 3). However, no systematic bias was detected in doxorubicin, intercalating between DNA bases, or in cisplatin, a DNA crosslinker creating adducts between purine residues. Hydroxyurea, impeding DNA repair by depleting deoxynucleotides, instead strongly favored haploids. Exposure to some mutagens increases the rate of ploidy switching [25]. To exclude confounding effects of mating type switching, we therefore quantified the stationary phase DNA content of haploid and diploid populations of five strains in the absence of stress and during Doxorubicin, Hydroxyurea and Cisplatin exposure. In no case was ploidy switching on the population level observed, although minor ploidy polymorphisms may have emerged in some cultures (Figure S4). Opposing ploidy effects were found also during nutrient restriction (Figure 2A, Figure 3). Depending on the nitrogen source, nitrogen restriction was either ploidy neutral, or favored either haploids or diploids. Thus, environments containing tryptophan or leucine as sole nitrogen sources provided advantages for diploidy whereas environments containing phenylalanine or urea as sole nitrogen sources benefitted haploids. Removal of essential micronutrients, forcing mobilization of internal nutrient storages across organelle surfaces, also alternately favored haploids (inositol depletion) or diploids (zinc, magnesium depletion) (Figure 2A). Also during exposure to harmful substances, the merits of ploidy shifted dramatically with the specific toxin encountered and failed to follow any of the hypothesized patterns. For example, exposure to Li^+^ strongly favored haploidy whereas no such bias was seen for Na^+^ (Figure 2A). This is remarkable given that these alkalic cations are considered to act intracellularly through similar mechanisms and are detoxified through similar cellular processes [26].

**Figure 4:**
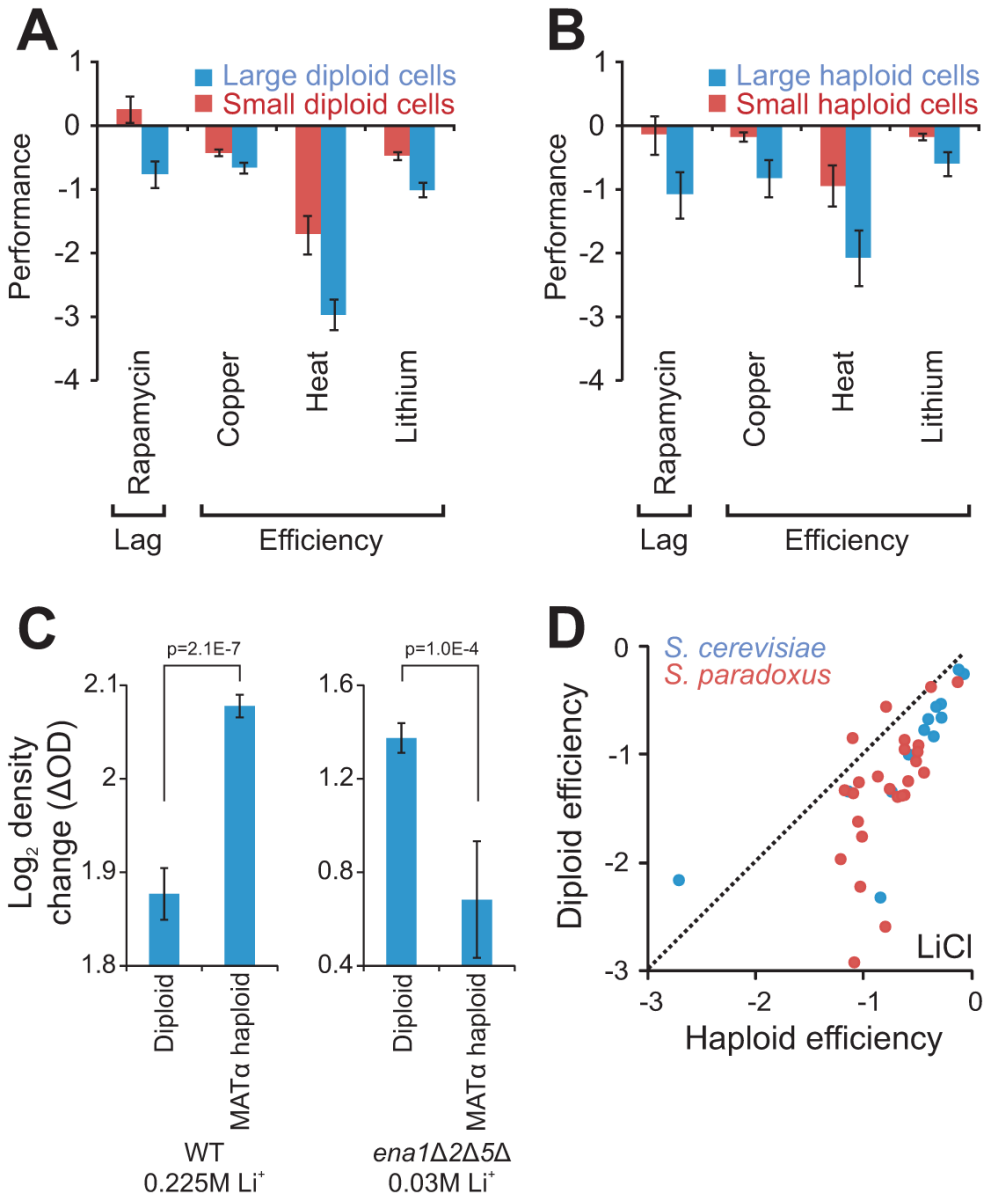
Cell size partially explains ploidy–environment interactions. A–B) Fitness components measures with a significant (FDR, α = 0.05) difference, both between large (n = 10) and small (n = 10) S288c haploids and between large (n = 29) and small (n = 20) S288c diploids. Large and small cells were constructed through individual deletion of different cell size defining genes. Note that data is shown on a log(2) scale. rror bars represent SEM. A) Performance of large and small diploid cells. B) Performance of large and small haploid cells. C) The tandem genes encoding the Li^+^ exporters *ENA1*,*2* and *5*, were deleted in the haploid S288c derivative BY4741 and the haploid deletion strain was autodiploidized through mating type switching. The total change in density (the efficiency) of mitotically reproducing populations exposed to 30 mM LiCl was obtained for *ena1*Δ*2*Δ*5*Δ haploids (n = 8) and diploids (n = 56) and compared to that of WT haploids (n = 16) and diploids (n = 16) in presence of 225 mM LiCl. Note that data is shown on a log(2) scale. Error bars represent SEM, p-values = Student's t-test. D) Growth efficiency of haploid and diploid versions of individual *S. cerevisiae* and *S. paradoxus* strains. Broken lines represent 1∶1 correlation (null hypothesis expectation).

In some cases, for example rapamycin and caffeine exposure, the picture was complicated by ploidy dependent trade-offs between the rate and efficiency of asexual proliferation. Most notably, when populations were supplied with an excess of nutrients and expanded at their maximal rate, *S. cerevisiae* haploids tended to reproduce faster asexually but achieved a lower total change in population density and were slower in initiating growth (Figure 4). Overall, our data falsified assumptions of generalizable effects of ploidy on mutation tolerance, toxin exposure and nutrient utilization, leading us to argue for more nuanced models based on the molecular architecture of cellular responses to individual ecological factors.

### Ploidy–environment interactions are partially explained by cell size

A shift from haploidy to diploidy often enlarges cell and organelle volume through prolonged repression of the G1 cyclin Cln3 which links cell cycle to cell size [27]. However, given that a roughly spherical form is maintained, such a volume increase is not accompanied by comparable enlargement of surface areas. Given an excess of nutrients other than glucose and no environmental stress, diploids of the universal reference strain S288c possess twice the cell volume of haploids, but their cell surface area is only 1.57 times larger. Diploids regulate their production of cell envelope proteins to match this distortion [28], but other protein classes are not as stringently regulated, suggesting a potential mechanism for ploidy-environment interactions. We reasoned that if ploidy-environment interactions indeed arise as consequences of cell size dependent distortions of volume-to-surface area ratios, then artificial cell size enlargement or reduction should inflict similar environment dependent shifts in asexual reproductive performance. Testing this prediction, S288c haploid and diploid yeasts artificially designed to have enlarged or reduced cell size through gene deletion [29] (Table S3), were cultivated in environments favoring either haploidy or diploidy in this particular cognate genetic background (Figure S5, Table S4). Of 24 mitotic fitness traits probed, 17% were clearly (FDR, α = 0.05) size dependent considering both haploids and diploids (Figure 4A, 4B). Small cells consistently showed shorter lag phase when exposed to rapamycin, a *Streptomyces* toxin inhibiting the growth-promoting TOR pathway. Small cells also showed consistently superior proliferation efficiency during exposure to heat, Cu^2+^ or Li^+^, presumably reflecting more efficient utilization of energy.

In S288c growth efficiency during Cu^2+^ or Li^+^ exposure, at the relevant pH (5.8), is almost completely determined by recent gene amplifications of the copper chelating metallothionein *CUP1* and Li^+^ exporter *ENA* [22]. Hence, the impact of ploidy and cell size on these traits was deemed likely to depend on copper chelation and lithium efflux respectively. To test if the ploidy effect on asexual growth efficiency during lithium exposure was indeed coupled to *ENA* mediated lithium efflux, the three S288c *ENA* genes *ENA1,2* and *5*, which derive from a single non-ancestral *ENA* variant recently introgressed from *S. paradoxus* into the European *S. cerevisiae* population and later amplified in tandem in S288c [22], were deleted in *MAT*α haploids and *MAT* ***a***/α diploids. Deletion of the *ENA* genes rendered both haploids and diploids hypersensitive to LiCl. Remarkably, when measuring the growth efficiency of Li^+^ exposed *ena1*Δ*2*Δ*5*Δ cells at 30 mM LiCl, causing a roughly similar trait reduction as that of WT cells exposed to 225 mM LiCl, we found the ploidy effect on efficiency to be not only obliterated but actually reversed by removal of the *ENA* genes (Figure 4C). Thus, when using the Ena genes for Li^+^ extrusion, haploids sustain a more efficient growth than diploids, whereas they when forced to rely on the lower capacity Nha1 system for Li^+^ extrusion [26] are overtaken by diploids. The superior mitotic efficiency of haploids when exposed to Li^+^ is conserved throughout *S. paradoxus* and *S. cerevisiae* (Figure 4D), regardless of the type and number of *ENA* genes maintained, suggesting this to be an evolutionary ancient trait. Interestingly, the slower growth rate of haploids when exposed to Li^+^, which appeared to be independent of cell size, was evident also in the absence of *ENA* genes (Figure S6). This disconnection between rate and efficiency of Li^+^ growth emphasizes the complexity of ploidy effects and the necessity to resolve mitotic fitness into its underlying components when considering the underlying molecular mechanisms.

### Ploidy–environment interactions are partially explained by mating type locus composition

**Figure 5:**
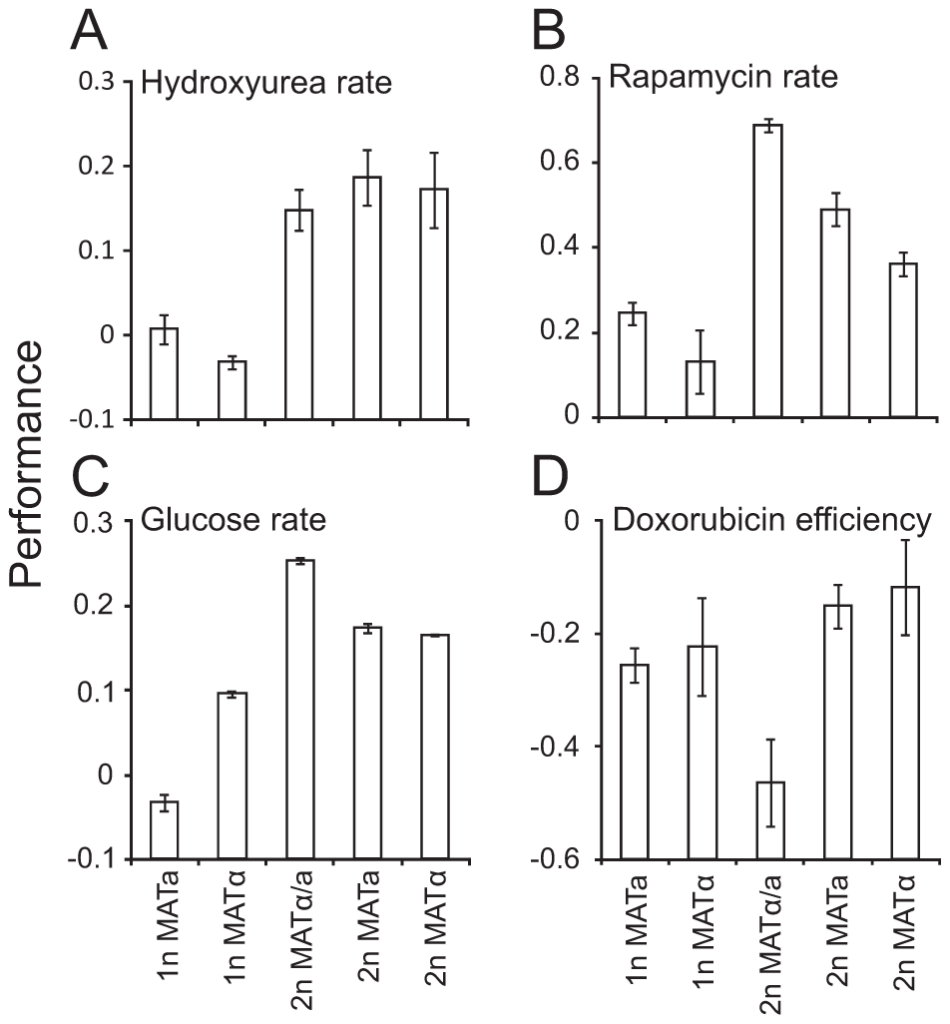
Mating type locus composition partially explains ploidy–environment interactions. Fitness component measures of S288c haploids, diploids heterozygotic, **a**/α, at the mating type locus and diploids hemizygotic, **a** or α, at the mating type locus, in various environments (n = 4). A) Mitotic growth rate in hydroxyurea, a sample environment where mating type locus composition fails to explain fitness differences between haploids and diploids. Note that data is shown on a log(2) scale. B–D) Environments in which fitness differences between ploidy states are partially or completely explained by mating type locus compositions (FDR, α = 0.05). Note that data is shown on a log(2) scale. B) Mitotic growth rate during rapamycin exposure C) Growth rate in nutrient excess and absence of stress D) Mitotic growth efficiency during doxorubicin exposure.

Diploids (2n) in yeast are naturally heterozygous at the mating-type locus (α/a), whereas haploids (1n) contain only one type of genetic information at this locus, either ***a*** or α. This single genetic difference underlies fundamental differences in life-cycle related phenotypes [30], and could explain the dramatic effects of ploidy on mitotic fitness components in different environmental contexts. To separate the effect of mating type from other ploidy effects, we considered S288c diploids that are hemizygous at the mating-type locus, carrying either α or ***a*** information. Together with the normal 2n (α/***a***), 1n (***a***) and 1n (α) strains, these were cultivated in environments favoring either S288c haploidy or diploidy (Table S4). Significant (FDR, α = 0.05) differences between the hemizygotic α and ***a*** diploids and the normal α/***a*** diploid, were then identified, pointing at cases in which the mating type locus contributed significantly to the trait differences between haploids and diploids. Most asexual proliferation traits, such as the atypical superior performance of S288c diploids in hydroxyurea, were completely independent of mating type locus composition (Figure 5A). 12.5% of the 24 tested traits were affected by mating type locus composition. This included the superior growth rate of S288c diploids in conditions of nutrient excess and absence of stress and the superior haploid efficiency of proliferation in the face of a doxorubicin mediated elevation of mutation rates (Figure 5B–5D). The superior growth rate of diploids following a challenge with the TOR inhibitor rapamycin effect is especially noteworthy given the cell size mediated beneficial impact of haploidy on rapamycin growth lag (Figure 4A, 4B). Thus, a diploid mating type enabled faster cell cycle progression during rapamycin exposure, whereas a haploid cell size enabled faster cell cycle re-entry in the same conditions. This underscores the complexity of the interplay between ploidy and environment. The TOR complexes function as key transcriptional activators of ribosomal gene expression [31]. Given that the strong and consistent elevation in ribosomal protein mRNA levels in haploids relative to diploids [32], the role of TOR in ribosomal protein transcription is a likely cause of the here observed ploidy effects.

## Discussion

### Ploidy–environment interactions have been conserved over long evolutionary time spans

*S. cerevisiae* lab strain gametes of complementary mating types mate and diploidize after only a few rounds of haploid clonal reproduction, thereafter maintaining diploid mitosis until nutrients in the local environment are exhausted [33]. Also in lab strain experimental evolutions, initially haploid populations sometimes end up as diploid through successive chromosome replications without cell division [34], [35]. This processes proceeds even when selection is limited through repeated single cell passages [36]. Thus, the drive towards diploidy has been considered to be deeply ingrained in the genome of yeast lab strains. Considering a large fraction of yeast genotypic space, we found no overall bias towards superior performance of diploids. The apparent discrepancy between the general tendency towards diploidization and the distinctly superior mitotic proliferation of haploids in many environments begs explanation. Yeast life history with frequent and narrow population bottlenecks promotes trait divergence through genetic drift [37] and it cannot be excluded that some of the observed ploidy effects represent non-beneficial traits that became fixed in the common ancestor of *S. cerevisiae* and *S. paradoxus* during periods of small population sizes. Furthermore, the routine approximation of yeast asexual reproduction to fitness [38] may not completely reflect the action of selection. Natural yeasts spend most of their chronological life time in non-dividing states, meaning a potentially superior fitness influence of viability. Viability is sometimes enhanced by spore form transitions [39], necessitating a preceding diploidization [19]. Conceivably, this could disconnect ploidy effects on asexual reproduction from ploidy effects on overall fitness. Nevertheless, the frequent conservation of ploidy effects across the 2 billion asexual generations separating *S. cerevisiae* and *S. paradoxus* suggests such a decoupling to be unlikely to explain the bulk of the observed effects. In fact, it implies strong selection to have acted on the ploidy-environments interactions in both these species since the time of their divergence. This leaves the alternative explanation; that the tendency towards diploidization is not a universal feature of *S. cerevisiae* in natural habitats. The recent emergence of yeast population genomics [21] and phenomics [22] has enforced the realization that *S. cerevisiae* properties vary within surprisingly wide boundaries. Ploidy preference, varying enormously between yeast species but unstudied over a wider section of the genotypic and ecological space of *S. cerevisiae*, may be similarly fleeting, as supported by a surprisingly large natural variation in ploidy at micro-ecological scales [40].

### Diploidy fails to confer a general asexual reproductive advantage during elevated mutation rates

Mutation rates are thought to be independent of ploidy state [41]; thus, an increase in DNA content confers a proportional rise in mutational load [42]. In addition, the almost universal lack of penetrance of gene-disrupting mutations as long as one functional copy remains [37], [43] causes mutation masking effects, impeding purging of mutations. Given the overwhelmingly negative nature of mutations, both these effects should favor haploidy in the long run. However, mutation masking may allow sustained proliferation during short periods of elevated mutation rates, selecting against haploidy in niches where such fluctuations are frequent [44]. Our data rejected a general asexual advantage of diploidy in environments elevating mutation rates. Applied doses of mutagens impaired the asexual proliferation of most strains. However, it cannot be excluded that these costs arose from perturbation of other cellular features, such as transcription, or were associated with drug export or metabolism, or arose from the costs of repairing DNA damage. Strictly speaking, we cannot tease apart the effects of unrepaired mutations, effects of repairing DNA damage, and effects on other cellular features. This calls for some caution when interpreting results. The influence of ploidy may also be strongly dependent on the type and mechanism of DNA damage. Double stranded breaks disproportionately challenge haploids as repair by homologous recombination, using an extra unperturbed chromosome copy, is by far the most efficient repair mode [45]. Smaller base lesions, resulting from oxidation and alkylating damage, impose no such requirements [46]. Haploid and diploid yeast activate different DNA repair pathways in response to replication stress imposed by Mcm4 impairment [47]. It is conceivable that e.g. the effect of ploidy on hydroxyurea tolerance, also impairing replication, may be due to this differential DNA repair activation. Increase in ploidy can also result in decreased genome stability due to disproportionate scaling of chromosome segregation components, notably the kinetochore, spindle and spindle pole body [48]. Such imbalances may fuel the strong effects of ploidy on cellular responses to gross chromosomal rearrangements [32] and affect the tolerance to mutagenic agents.

A natural shift from haploidy to diploidy also alters mating type locus composition, from *MAT* ***a*** or *MAT*α to *MAT* ***a***/*MAT*α. This mediates a shift from haploid to diploid specific transcription programs and from preparedness to mate to readiness to pass through meiosis and later sporulation [49]. The affected pathways are often pleiotropic, raising the potential for phenotypic hitchhiking of mitotic ploidy effects with effects on mating, meiosis and sporulation. The shift from axial budding in haploids to bipolar budding in diploids is one potential mechanistic mediator of such pleiotropic consequences [50], as is the 10-fold increase in transposon production in haploids resulting from induction of the pheromone signalling pathway [28], [51]. We found mating type locus composition to account for 12.5% of ploidy effects on mitotic properties in S288c. This included superior haploid asexual reproductive efficiency following exposure to doxorubicin. Doxorubicin induces double strand breaks which requires repair through homologous recombination or non-homologous end joining. The latter process is turned off in *MAT* ***a***/*MAT*α S288c diploids through the ***a***1-α2 repression of the *NEJ1* transcription factor [52], [53], suggesting a likely molecular cause for ploidy dependent doxorubicin tolerance in S288c.

### Cell size and mating type locus composition confer environment-specific asexual reproductive advantages manifesting as ploidy effects

An increase in ploidy often has similar effects on cell size through repression of the G1 cyclin linking cell cycle progression to cell size [27]. Accordingly, benefits of increased ploidy could arise in toxic environments due to elevated cell volume-to-surface area ratios, reducing uptake of harmful compounds relative detoxification capabilities given that the latter are volume dependent [18]. Analogously, microhabitats where fitness is constrained primarily by nutrient accessibility may favor haploidy due to the lower volume-to-surface area ratio and enhanced nutrient uptake relative to volume [15]–[17]. This presupposes nutrient transport across membranes to be a limiting factor in the utilization of the nutrient, which often, but not always, appears to be the case [15]. The scope of the current study and the absence of a consistent effect of ploidy on either toxin tolerance or nutrient utilization provide grounds for rejecting both these hypotheses in their most generalized form. A potential cause of the failure of these hypotheses is the ploidy dependent regulation of cell size in response to environmental cues. In nutrient rich environments diploid S288c boasts 1.57 times the volume of haploids but carbon restriction completely eliminates this difference [15]. Furthermore, it is doubtful whether substance influx is the sole, or even may, variable affecting asexual reproduction that is altered by cell size. Both efflux and vacuolar storage often have substantial impacts on yeast proliferation under nutrient restriction and toxin exposure and these may be similarly dependent on volume-to-surface area ratios. Nevertheless, individual ploidy-environment interactions were sometimes explained by cell size. In the case of the superior asexual reproductive efficiency of haploids during exposure to Li^+^, we traced these effects to the presence of the Ena lithium pumps. Given the enormous influence of the *ENA* locus on asexual reproductive efficiency in lithium environments [22] and the belief that ATP driven pumping of Li^+^ by Ena proteins completely controls Li^+^ efflux at intermediate [H^+^] [26], this was not entirely surprising. In absence of the Ena transporters, yeast is forced to rely on Nha1 for alkali metal efflux, a pump that has a vastly lower capacity at pH 5.8 as it is driven by proton influx [26]. Interestingly, the more efficient growth of haploids during Li^+^ exposure was not only obliterated by removal of the *ENA* genes, but reversed, now favoring diploids. This suggests that ploidy has reverse impacts on the Nha1 and the Ena systems, illustrating how a simple molecular shift can completely alter the relative merits of haploidy and diploidy in a particular environmental context. This is consistent with a recent finding that adaptive mutations emerging and driving towards fixation in evolving laboratory populations have different effect sizes when reconstituted individually in haploid and diploid genomic contexts [54]. Ena2, the Ena variant with highest affinity for Li^+^, appears to be largely unregulated and expressed at basal levels [55], suggesting that the density of Ena2 in the diploid membrane, which presumably has a higher surface area, may be lower than the density in the haploid membrane. This may explain the ploidy effects.

Although alterations in cell volume-to-surface area ratios may mediate many cell size dependent ploidy-environments interactions, it should be noted that also organelle volume-to-surface area ratios fluctuate as a function of cell size and environmental context. Expansions and fragmentations of yeast vacuoles [56], and expansion of the nucleus [57] are well documented examples. Furthermore, a host of other biochemical and regulatory properties also depend on cell size [15], [29], such as silencing at some subtelomeric regions via unknown posttranscriptional mechanisms [58]. All these may contribute to ploidy-environment interactions affecting mitotic properties. Yeast mitotic properties in different environmental contexts also tend to be highly polygenic [59], [60], increasing the likelihood that detected ploidy effects may be composites of cell size, mating type and DNA content influences. This enhances the challenge of molecularly decoding ploidy dependent traits and may explain why 70% of S288c ploidy effects could not be accounted for by considering cell size and mating type individually. Overall, our findings revealed an unsuspected prevalence of ploidy effects in yeast and suggested a dynamic interplay between ploidy and environment, involving evolutionary trade-offs of surprisingly ancient origin and diverse molecular roots.

## Materials and Methods

### Yeast strains and population growth experiments

24 *S. cerevisiae* and 27 *Saccharomyces paradoxus* isolates, corresponding to known yeast populations, geographic origins and source environments (Table S1), were isolated as described [21]. Following deletion of *URA3* (KanMX) and *HO* (HygMX), mating and sporulation, haploid (*MAT* ***a*** and *MAT*α) and autodiploid (*MAT* ***a***/*MAT*α) were obtained [61] and long-time stored at −80°C in 20% (w/v) glycerol. Strains were subjected to high throughput phenotyping by micro-cultivation in 33 environments essentially as previously described [62], [63]. A complete list of environments can be found in Table S2. Strains were inoculated in 350 µL of Synthetic Defined (SD) medium (0.14% yeast nitrogen base, 0.5% ammonium sulfate and 1% succinic acid; 2% (w/v) glucose; 0.077% Complete Supplement Mixture (CSM, ForMedium), pH set to 5.8 with NaOH or KOH) and incubated for 48 h at 30°C. For experiments where the removal of a specific nutrient was studied, the pre-culture was performed in absence of this nutrient in order to deplete intracellular storages. For experiments where alternative nitrogen sources were used, two consecutive pre-cultures were performed, the first in limiting concentrations of ammonium, 29 µg N/mL, in order to avoid excessive nitrogen storage, the second replacing ammonium with the indicated nitrogen source in amounts corresponding to an equivalent number of nitrogen atoms. Except for the nitrogen source indicated and 20 mg/L uracil, which cannot be used as sole nitrogen source [22], no other nitrogen was supplied in these experiments. For experimental runs, precultures were diluted 35× to an OD of 0.03–0.15 in 350 µL of SD medium and cultivated for 72 h in a Bioscreen analyzer C (Growth curves Oy, Finland). Optical density was measured using a wide band (450–580 nm) filter. Incubation was at 30.0°C (±0.1°C) with ten minutes preheating time. Plates were subjected to shaking at highest shaking intensity with 60 s of shaking every other minute. OD measurements were taken every 20 minutes.

### Extraction of mitotic fitness components

The rate (population doubling time), lag (population adaptation time) and efficiency (total change in population density) of asexual reproduction were extracted from high density growth curves and log_2_ transformed [62], [63]. Relative mitotic fitness components for each strain and environment, LSC*_ij_*, were calculated by normalization of each measurement to an internal (WT) standard (haploid S288c, *MAT*α, n = 8) as:
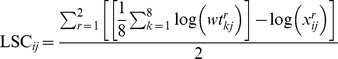

*wt_kj_* is the trait measure of the *k* ^th^ measurement of the wild type for trait *j*, *x_ij_* is the measure of strain *i* for trait *j* and *r* indicates the run. To maintain directionality between the mitotic fitness components, the measure for proliferation efficiency was inverted. Note that the lag measures generally should be treated with caution due to its higher sensitivity to bias. For example, it cannot be excluded that some early growth is misclassified as a lag, due to the cell density increase being below the threshold of detection.

### Ploidy effects

To compare haploid and diploid asexual proliferative capacity, a mean of the two mating types (each n = 2) was used to derive a single measure of haploid performance. This was compared to that of the diploid (n = 4). For S288c, a substantially higher number of *MAT*α haploids (n = 32), *MAT* ***a*** haploids (n = 16) and diploids (n = 16) were tested. Ploidy effects were calculated as the mean difference between haploid and diploid phenotypes. Statistical significance of trait differences between haploids and diploids was tested using a two-tailed homoscedastic Student's t-test False Discovery Rates (α = 0.05) were applied to account for multiple hypotheses testing [64]. Homoscedastic Student's t-test and False Discovery rate corrections were similarly used for all two-group comparison situations, except were otherwise mentioned (see below). Note that the four different replicates of haploids and diploids were placed in two different well positions in four different plates which were run in two different Bioscreen instruments, hence accounting for much of the spatial bias of well position. The normalization to eight different internal standards per plate also almost completely removes temporal, batch-based, instrument based and plate based bias. Despite these measures, some bias is unavoidable, meaning that we are likely to underestimate the true uncertainty. Hence, the true number of false positives is likely to be somewhat higher than 1 in 20 positive calls and displayed error bars are likely to be slightly overoptimistic.

### Clustering

Hierarchical clustering, as outlined in [65], was performed using a centered Pearson correlation coefficient. Group clustering was achieved using group averages. Missing measurements were treated as “missing data”.

### Analysis of variance

Similarities between pairs of yeast strains was calculated similarly for ploidy effects and haploid and diploid phenotypes. The similarity between two strains was calculated as the Pearson correlation coefficients (r^2^) between strains, after omitting missing values and after scaling phenotype values across strains to unit variance. In order to quantify the contribution of species divergence, population structure and source environments to the observed variation in such strains we performed one-way analysis of variance (ANOVA) with the function lm in R. In three separate analyses, we assumed equal variances and tested (F-test) for differences in the means for pairs of strains (i) between and within species, (ii) between and within population, (iii) and between and within source environment. For significant effects, we used adjusted r^2^ values to quantify the explained variance.

### Effect of cell size on ploidy–environment interactions

Large (n = 10) and small (n = 10) haploid strains and large (n = 29) and small (n = 20) diploid strains (Table S3) were cultivated as two independent replicates in a subset of environments (Table S4) as described above. Strains corresponded to single gene deletions (*gene x::kanMX6*) in the S288c derivatives BY4741 and BY4743 (http://www-sequence.stanford.edu/group/yeast_deletion_project/deletions3.html) and were previously determined as being cell size extremes [29]. Growth data was analyzed as described above. The performance difference between large and small cells was independently tested for haploids and diploids; a significant (FDR, α = 0.05) difference for both haploids and diploids was required for positive calls.

### Effect of mating type locus composition on ploidy–environment interactions

To obtain diploid S288c hemizygotic at the mating type locus, *MAT* ***a*** and *MAT*α respectively were individually deleted in a/α diploid S288c, start to stop, using a *KanMX6* cassette as described [66]. *MAT* ***a*** and *MAT*α haploids and the *MAT* ***a***, *MAT*α and *MAT* ***a***/*MAT*α diploids (all n = 4) were pre-cultivated and cultivated in a subset of environments (Table S4) and analyzed as described above. We cannot completely exclude the possibility that hemizygosity at the mating type locus *per se* affects the phenotypes measured. However, there is a strong general tendency of yeast hemizygotes to mimic the corresponding homozygotes [67], suggesting that such confounding effects are unlikely.

### Effects of Ena proteins on ploidy–lithium interactions

A haploid derivate of S288c, BY4741, lacking *ENA1,2* and *5*, was provided by Hana Sychrova. A diploid version of this strain was obtained by transformation with an HO plasmid containing a *URA3* marker, selection of 14 *MAT* ***a*** */*α diploid spores on –uracil media and verification through PCR directed at the mating type locus and PCR product size analysis on gel. Despite repeated tries, diploids could not be coerced to sporulate. *MAT* **a** haploids could therefore not be obtained. MATα haploids (n = 8) and *MAT* ***a***/*MAT*α diploids (four replicates of each spore, n = 56) were pre-cultivated and cultivated in conditions with and without LiCl. As the deletion of *ENA1,2* and *5* renders cells hypersensitive to, [LiCl] was reduced to 30 mM in order to obtain a reduction of mitotic fitness roughly comparable to that observed in WT cells exposed to 0.225M LiCl.

### Quantification of stationary phase DNA content

Quantification of DNA content by flow cytometry was carried out using propidium iodide (PI) staining as described [68], [69] with some modifications. Cells were grown in Synthetic Defined (SD) medium (as above) with and without 20 µg/mL doxorubicin, 100 µg/mL cisplatin or 15 mg/mL hydroxyurea and incubated for 48 h at 30°C. Approximately 1×10^7^ cells were recovered by centrifugation and washed with 1 mL of PBS buffer (8 g/L NaCl, 0.2 g/L KCl, 1.44 g/L Na_2_HPO_4_, 0.24 g/L KH_2_PO_4_, pH 7,4). Cells were fixed with 1 mL of cold 70% ethanol and incubated 1 h at room temperature. After washing with PBS buffer, cells were resuspended in 300 µL of 50 mM sodium citrate containing 0.1 mg/mL RNAse A and incubated overnight at 37C. Finally, cells were washed and resuspended in 500 µL of PBS buffer and sonicated to disrupt aggregates (3×10 s). 2 µL of 1 µg/µL PI was added to each sample and incubated at 37C for 20 min. DNA content was determined using a FACSAria cytometer (BD Bioscience). Counting in total 10.000 events (cells), the number of events (cells) as a function of signal intensity (DNA content) was determined using the 488 nm line of an argon-ion laser for PI excitation and reading the PI emission at 578 nm.

## Supporting Information

Figure S1 Ploidy–environments interactions are as likely to favor haploidy and diploidy, independent of population or source environment. The overall asexual performance of haploids and diploids from distinct populations and source habitats was compared. All environments were considered but each mitotic fitness component was investigated separately. No significant general difference between the two ploidy states (FDR, α = 0.05) were found considering any population or source environment. Note that data is shown on a log(2) scale. Error bars represent SEM. A) Population B) Source habitat.
(PDF)

Figure S2 Ploidy–environments interactions are as likely to favor haploidy and diploidy, independent of strain. The overall asexual reproductive performance of haploids and diploids from distinct genetic backgrounds was compared. All environments were considered but each mitotic fitness component was investigated separately. No significant general difference between the two ploidy states (FDR, α = 0.05) were found considering any strain. Note that data is shown on a log(2) scale. Error bars represent SEM.
(PDF)

Figure S3 Trade-offs between yeast ploidy states in optimal environments. Performance of haploid (n = 4) and diploid (n = 2) versions of individual *S. cerevisiae* (blue) and *S. paradoxus* (red) strains in an environment with nutrient excess and no stress where the yield is limited by the amount of glucose (2%) that is present. Broken lines represent a 1∶1 correlation (null hypothesis expectation). Note that data is shown on a log(2) scale.
(PDF)

Figure S4 Short-term proliferation in presence of DNA damage inducing agents does not alter ploidy states of haploid or diploid populations. The relative number of cells with a particular DNA content was quantified for haploid and diploid populations of *S. cerevisiae* strains L-1528, Y12, DBVPG6765 and UWOPS83-783.3 and *S. paradoxus* strain N-44, after cultivation in absence and presence of 20 µg/mL doxorubicin, 100 µg/mL cisplatin or 15 mg/mL hydroxyurea. DNA of stationary phase cultures were stained with propidium iodide (PI) and analyzed by FACS cytometry, counting 10.000 events (cells). The number of events (cells) as a function of signal intensity (DNA content) was determined. Peak positions of haploid and diploid populations, corresponding to G1 and G2 phases with replicated and non-replicated DNA respectively, are indicated (arrows). Note that DBVPG6765 cells are highly sensitive to doxorubicin and largely arrested in G2, explaining the absence of G1 peak. Correspondingly, N-44 cells are highly sensitive to cisplatin. Partial arrest in different phases can also be seen for other populations, accounting for much of strain variations in relative heights of G1 and G2 peaks for different strains.
(PDF)

Figure S5 Ploidy–environment interactions in the S288c reference strain. Fitness traits with a significant (FDR, α = 0.05) difference between haploids (n = 48) and diploids (n = 16) of the *S. cerevisiae* universal reference strain S288c. Note that data is shown on a log(2) scale. Error bars represent SEM.
(PDF)

Figure S6 The ploidy dependence of the mitotic growth rate during Li^+^ exposure is independent of the main Li^+^ exporter Ena. The three tandemly amplified *ENA* genes, *ENA1*,*2* and *5*, was deleted in the S288c derivative BY4741. The haploid deletion strain was autodiploidized through mating type switching. The resulting *ena1*Δ*2*Δ*5*Δ haploids and diploids showed a vast increase in population doubling time relative the WT during Li^+^ exposure, necessitating a substantial reduction in [LiCl]. WT haploids (n = 16) and diploids (n = 16) and *ena1*Δ*2*Δ*5*Δ haploids (n = 8) and diploids (n = 56) were microcultivated in 0.225M and 0.03M LiCl respectively and population doubling times were extracted. Note that data is shown on a log(2) scale. Error bars = SEM. P-values correspond to a homoscedastic, two-tailed Student's t-test. The ploidy dependence of the doubling time during Li^+^ exposure was unaffected by removal of the *ENA* genes, diploids growing significantly faster than haploids. Hence, the ploidy effect of Li^+^ on mitotic growth rate is independent of the extrusion of Li^+^ via the *ENA* genes.
(PDF)

Table S1 Natural yeast isolates used in the study. “Population” refers to which of five *S. cerevisiae* or three *S. paradoxus* clean populations strain belongs to. For mosaic strains (containing genetic information from more than one population), the population donating the majority of the genetic information is indicated. “Source” refers to the source environment from which the strain was originally isolated. For YS9 (*MAT* ***a***), and 322134S (*MAT* ***a***), only one mating type was tested.
(DOC)

Table S2 Environments used in the screen. “Carbon source” indicates that 2% glucose was substituted with the indicated concentration of the relevant carbon source. “Nitrogen source” indicates that 0.5% ammonium sulfate was substituted with the relevant nitrogen sources at nitrogen limiting concentrations (corresponding to 29 µg N/mL). Except for the addition of 20 mg/L uracil, which cannot be used as nitrogen source by any of the strains, no other nitrogen was supplied. Furthermore, two consecutive pre-cultures were performed to deplete internal storages of nitrogen; the first was performed using nitrogen limiting amounts of ammonium sulfate, the second using nitrogen limiting amounts of the indicated nitrogen source. “Nutrient depletion” indicates that experiments were performed in medium completely lacking the indicated nutrient.
(DOC)

Table S3 Gene deletion strains used to screen for cell size effects on mitotic performance. Single gene deletions in the BY4741 (haploid) or, as homozygotes or heterozygotes, in the BY4743 (diploid) background used to screen for effects of cell size on mitotic performance in different environmental contexts (see Table S4).
(DOC)

Table S4 Environments tested in cell size and mating type locus experiments.
(DOC)
