# Supplementary figures and images for: Ancient Evolutionary Trade-Offs between Yeast Ploidy States
Source: PLoS Genet. 2013 Mar 21;9(3):e1003388. doi: 10.1371/journal.pgen.1003388 (PMC3605057; doi:10.1371/journal.pgen.1003388)

A

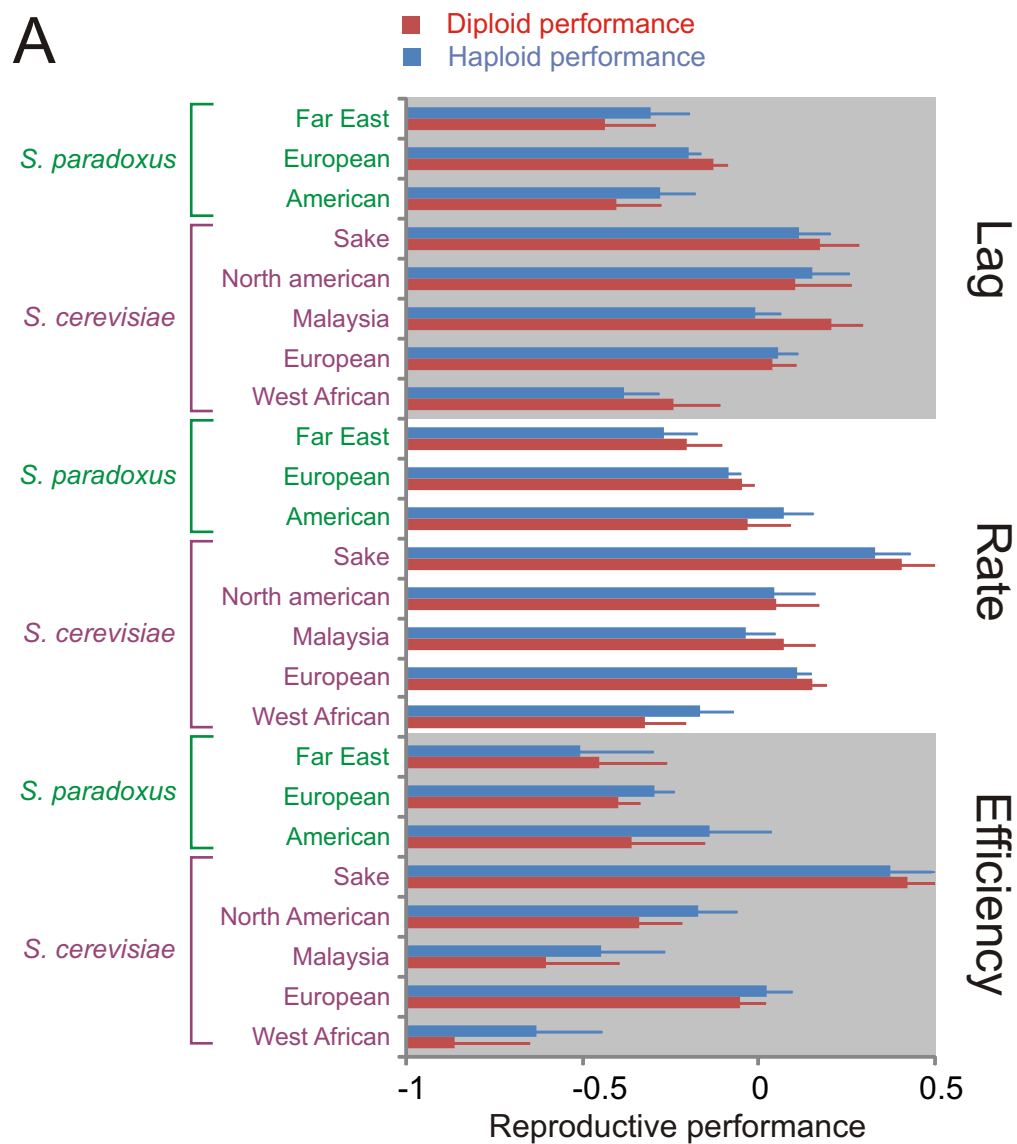

B

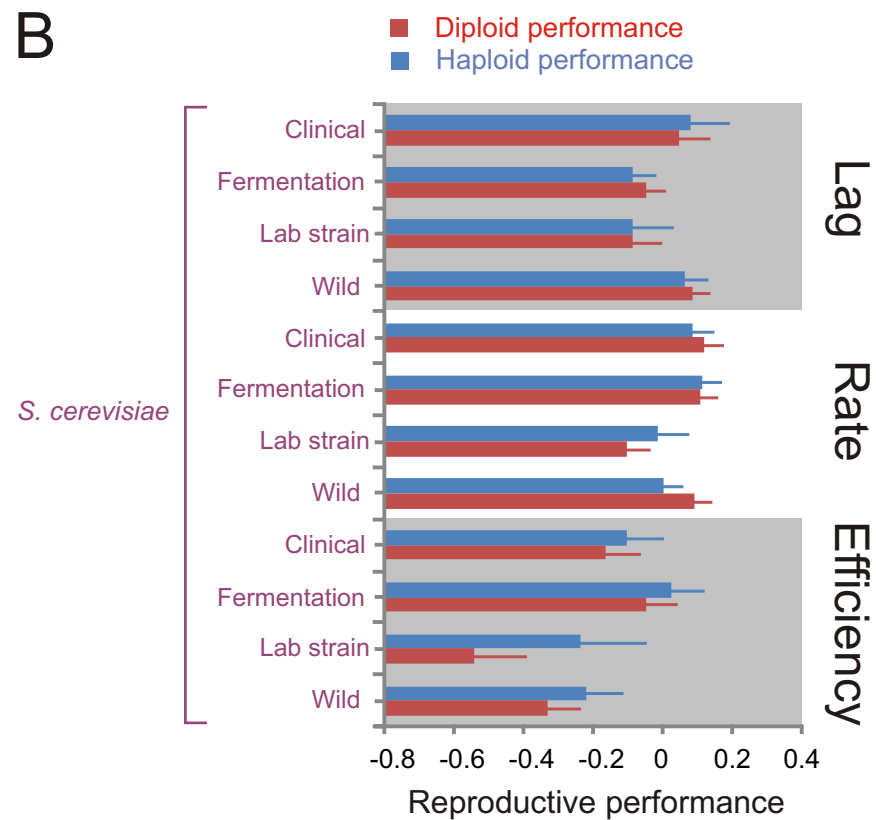

Supplement: Figure S1 — Ploidy–environments interactions are as likely to favor haploidy and diploidy, independent of population or source environment. The overall asexual performance of haploids and diploids from distinct populations and source habitats was compared. All environments were considered but each mitotic fitness component was investigated separately. No significant general difference between the two ploidy states (FDR, α = 0.05) were found considering any population or source environment. Note that data is shown on a log(2) scale. Error bars represent SEM. A) Population B) Source habitat. (PDF) [file pgen.1003388.s001.pdf]

## Glucose growth

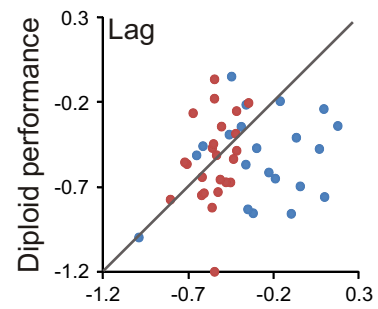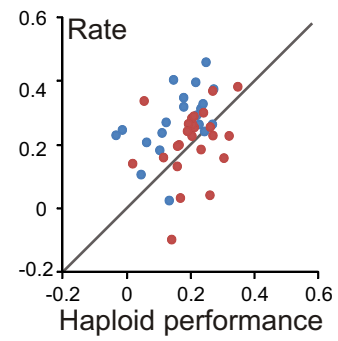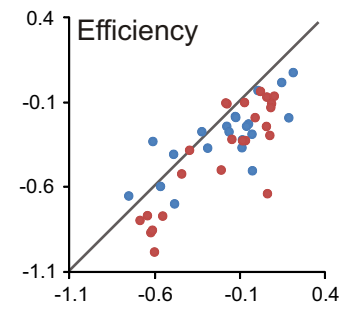

Supplement: Figure S3 — Trade-offs between yeast ploidy states in optimal environments. Performance of haploid (n = 4) and diploid (n = 2) versions of individual S. cerevisiae (blue) and S. paradoxus (red) strains in an environment with nutrient excess and no stress where the yield is limited by the amount of glucose (2%) that is present. Broken lines represent a 1∶1 correlation (null hypothesis expectation). Note that data is shown on a log(2) scale. (PDF) [file pgen.1003388.s003.pdf]

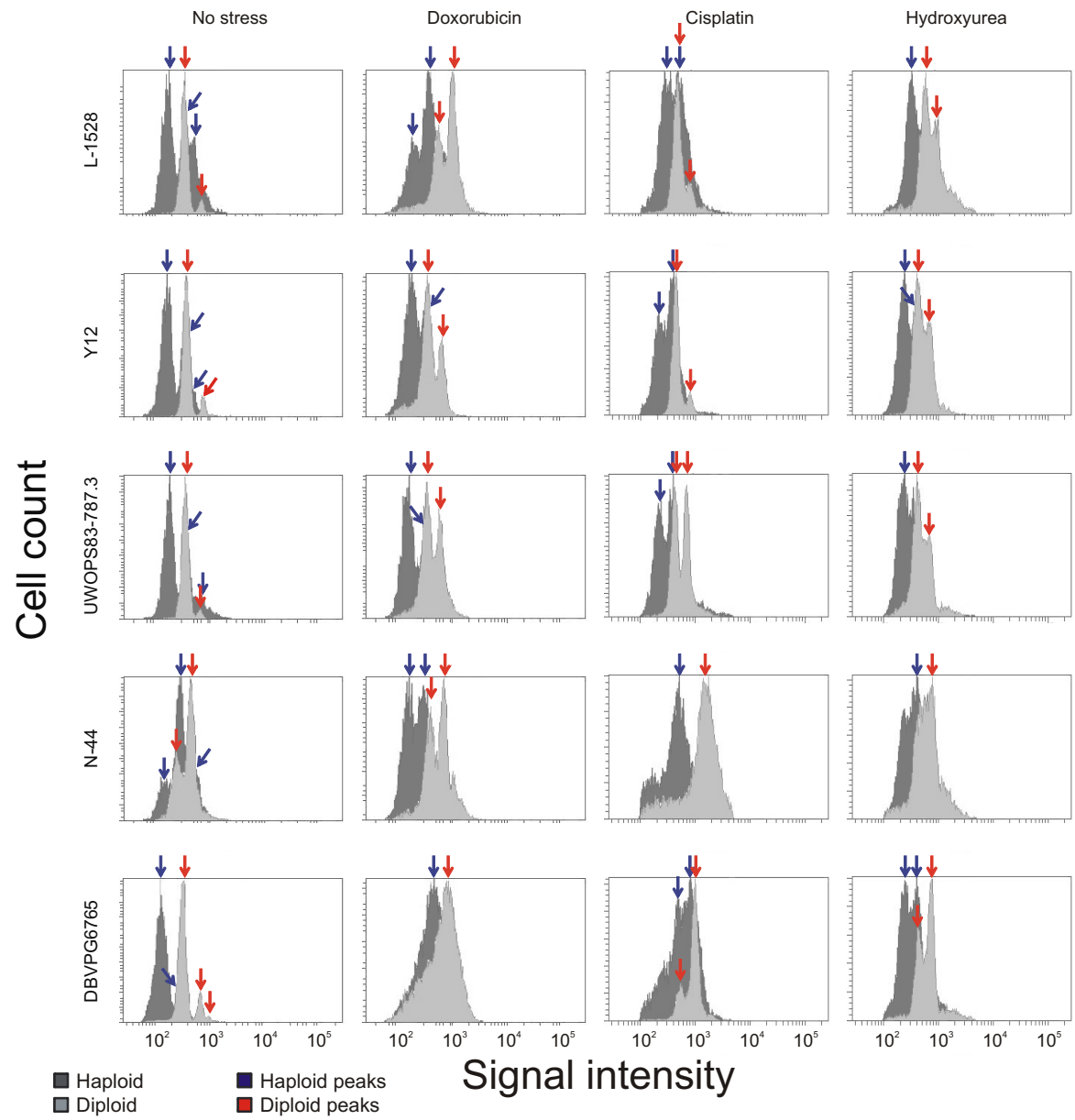

Supplement: Figure S4 — Short-term proliferation in presence of DNA damage inducing agents does not alter ploidy states of haploid or diploid populations. The relative number of cells with a particular DNA content was quantified for haploid and diploid populations of S. cerevisiae strains L-1528, Y12, DBVPG6765 and UWOPS83-783.3 and S. paradoxus strain N-44, after cultivation in absence and presence of 20 µg/mL doxorubicin, 100 µg/mL cisplatin or 15 mg/mL hydroxyurea. DNA of stationary phase cultures were stained with propidium iodide (PI) and analyzed by FACS cytometry, counting 10.000 events (cells). The number of events (cells) as a function of signal intensity (DNA content) was determined. Peak positions of haploid and diploid populations, corresponding to G1 and G2 phases with replicated and non-replicated DNA respectively, are indicated (arrows). Note that DBVPG6765 cells are highly sensitive to doxorubicin and largely arrested in G2, explaining the absence of G1 peak. Correspondingly, N-44 cells are highly sensitive to cisplatin. Partial arrest in different phases can also be seen for other populations, accounting for much of strain variations in relative heights of G1 and G2 peaks for different strains. (PDF) [file pgen.1003388.s004.pdf]

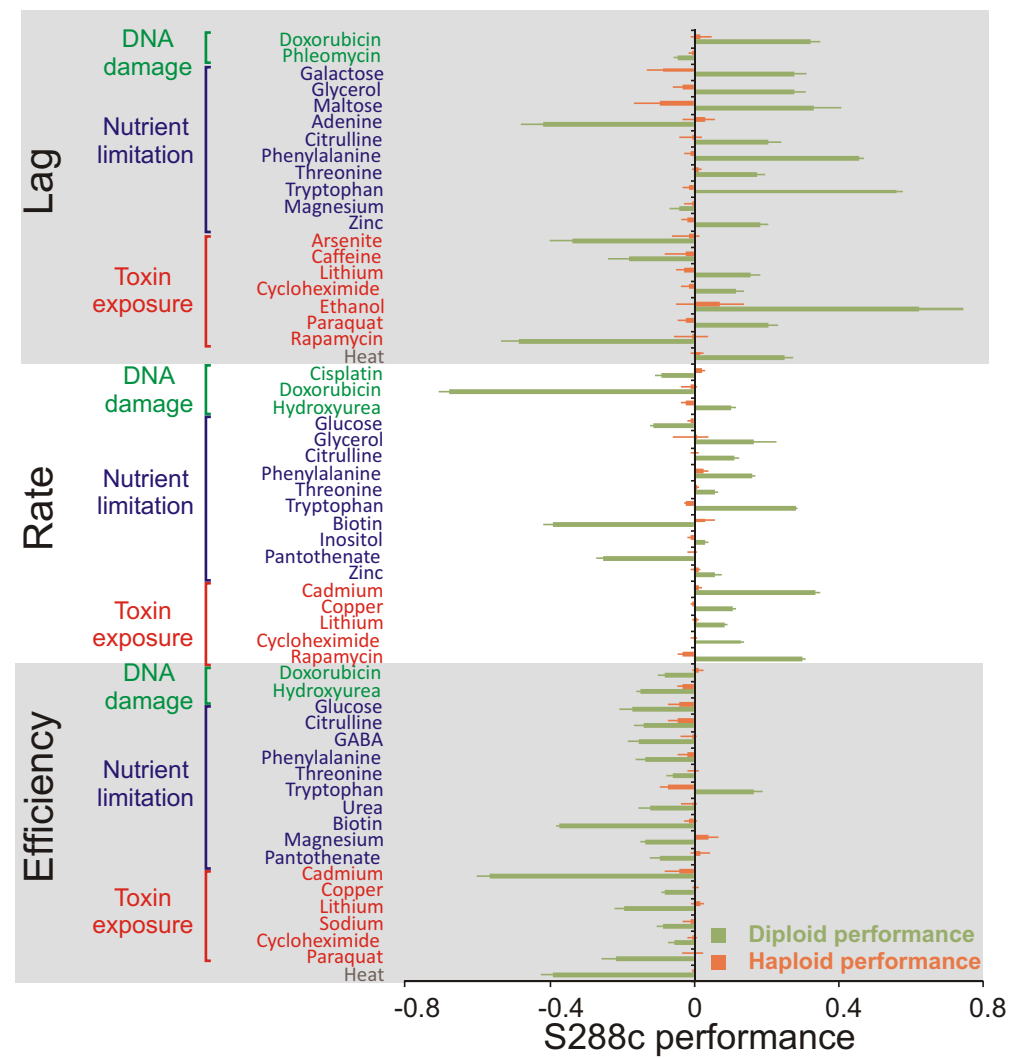

Supplement: Figure S5 — Ploidy–environment interactions in the S288c reference strain. Fitness traits with a significant (FDR, α = 0.05) difference between haploids (n = 48) and diploids (n = 16) of the S. cerevisiae universal reference strain S288c. Note that data is shown on a log(2) scale. Error bars represent SEM. (PDF) [file pgen.1003388.s005.pdf]

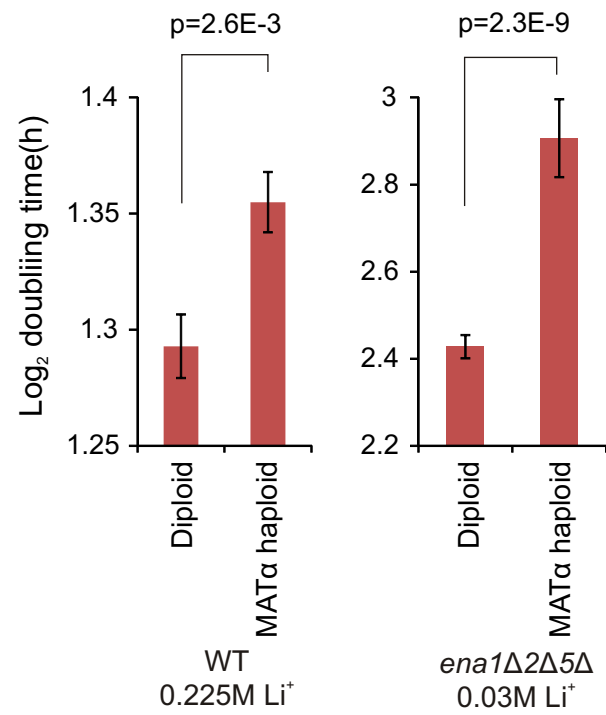

Supplement: Figure S6 — The ploidy dependence of the mitotic growth rate during Li+ exposure is independent of the main Li+ exporter Ena. The three tandemly amplified ENA genes, ENA1,2 and 5, was deleted in the S288c derivative BY4741. The haploid deletion strain was autodiploidized through mating type switching. The resulting ena1Δ2Δ5Δ haploids and diploids showed a vast increase in population doubling time relative the WT during Li+ exposure, necessitating a substantial reduction in [LiCl]. WT haploids (n = 16) and diploids (n = 16) and ena1Δ2Δ5Δ haploids (n = 8) and diploids (n = 56) were microcultivated in 0.225M and 0.03M LiCl respectively and population doubling times were extracted. Note that data is shown on a log(2) scale. Error bars = SEM. P-values correspond to a homoscedastic, two-tailed Student's t-test. The ploidy dependence of the doubling time during Li+ exposure was unaffected by removal of the ENA genes, diploids growing significantly faster than haploids. Hence, the ploidy effect of Li+ on mitotic growth rate is independent of the extrusion of Li+ via the ENA genes. (PDF) [file pgen.1003388.s006.pdf]
