# Supplementary material for: Ancient Evolutionary Trade-Offs between Yeast Ploidy States
Source: PLoS Genet. 2013 Mar 21;9(3):e1003388. doi: 10.1371/journal.pgen.1003388 (PMC3605057; doi:10.1371/journal.pgen.1003388)
Supplement: Figure S2 — Ploidy–environments interactions are as likely to favor haploidy and diploidy, independent of strain. The overall asexual reproductive performance of haploids and diploids from distinct genetic backgrounds was compared. All environments were considered but each mitotic fitness component was investigated separately. No significant general difference between the two ploidy states (FDR, α = 0.05) were found considering any strain. Note that data is shown on a log(2) scale. Error bars represent SEM. (PDF) [file pgen.1003388.s002.pdf]

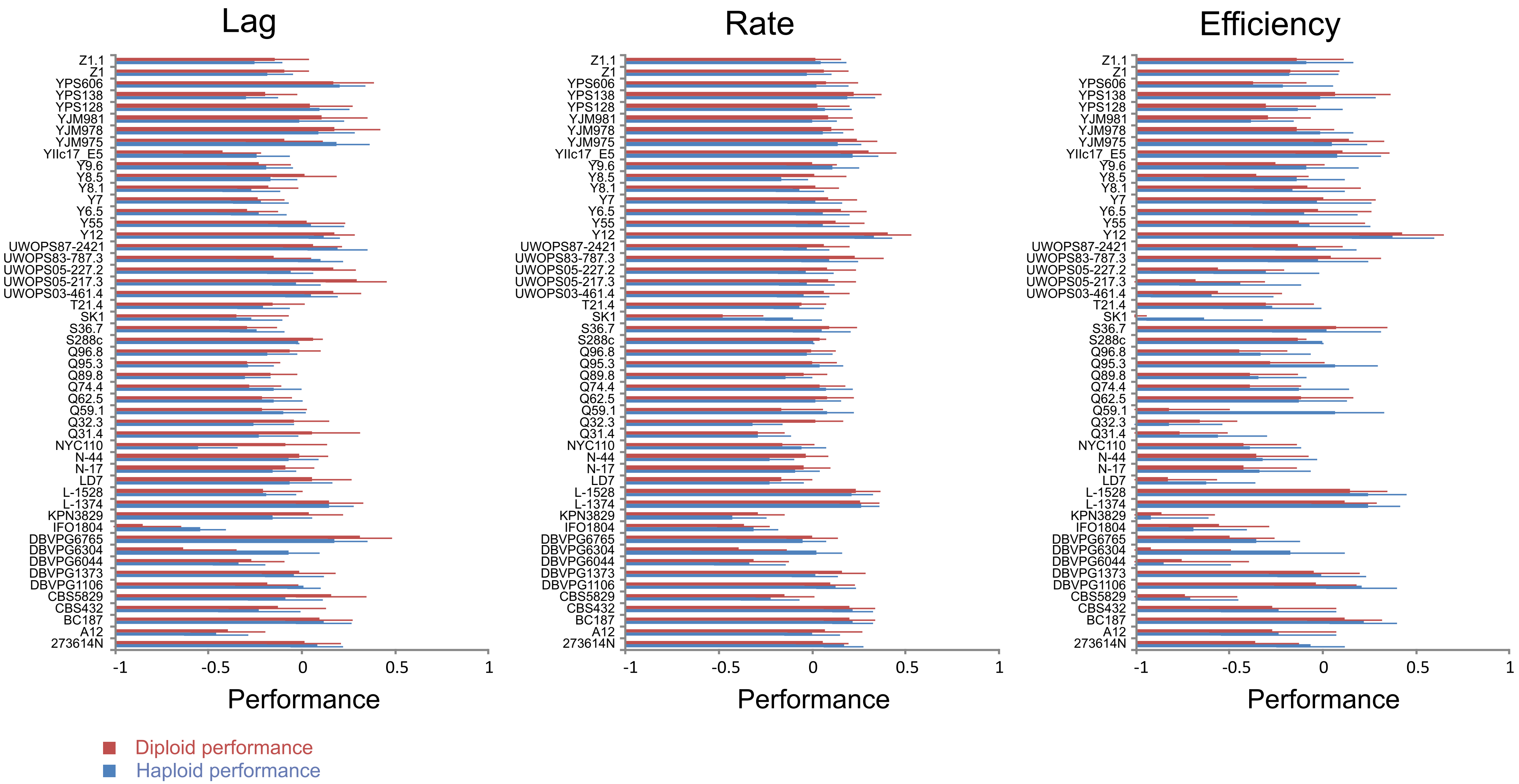
Lag
Rate
Efficiency
Z1.1
Z1
YPS606
YPS138
YPS128
YJM981
YJM978
YJM975
YIIc17_E5
Y9.6
Y8.5
Y8.1
Y7
Y6.5
Y55
Y12
UWOPS87-2421
UWOPS83-787.3
UWOPS05-227.2
UWOPS05-217.3
UWOPS03-461.4
T21.4
SK1
S36.7
S288c
Q96.8
Q95.3
Q89.8
Q74.4
Q62.5
Q59.1
Q32.3
Q31.4
NYC110
N-44
N-17
LD7
L-1528
L-1374
KPN3829
IFO1804
DBVPG6765
DBVPG6304
DBVPG6044
DBVPG1373
DBVPG1106
CBS5829
CBS432
BC187
A12
273614N
-1
-0.5
0
0.5
1
Performance
Diploid performance
Haploid performance
