# Supplementary material for: Ancient Evolutionary Trade-Offs between Yeast Ploidy States
Source: PLoS Genet. 2013 Mar 21;9(3):e1003388. doi: 10.1371/journal.pgen.1003388 (PMC3605057; doi:10.1371/journal.pgen.1003388)
Supplement: Table S1 — Natural yeast isolates used in the study. “Population” refers to which of five S. cerevisiae or three S. paradoxus clean populations strain belongs to. For mosaic strains (containing genetic information from more than one population), the population donating the majority of the genetic information is indicated. “Source” refers to the source environment from which the strain was originally isolated. For YS9 (MAT a), and 322134S (MAT a), only one mating type was tested. (DOC) [file pgen.1003388.s007.doc]

**Table S1** **Natural yeast isolates** **used in the study**

“Population” refers to which of five *S. cerevisiae* or three *S. paradoxus* clean populations strain belongs to. For mosaic strains (containing genetic information from more than one population), the population donating the majority of the genetic information is indicated. “Source” refers to the source environment from which the strain was originally isolated. For YS9 (MATa), and 322134S (MATa), only one mating type was tested.

| **Name** | **Species** | **Population** | **Source** |
| --- | --- | --- | --- |
| **S288c** | *S. cerevisiae* | Mosaic | Lab |
| **Y55** | *S. cerevisiae* | Mosaic, African | Lab |
| **SK1** | *S. cerevisiae* | Mosaic, African | Lab |
| **YJM978** | *S. cerevisiae* | European | Clinical |
| **YJM981** | *S. cerevisiae* | European | Clinical |
| **YJM975** | *S. cerevisiae* | European | Clinical |
| **322134S** | *S. cerevisiae* | Mosaic, European | Clinical |
| **273614N** | *S. cerevisiae* | Mosaic, European | Clinical |
| **DBVPG1373** | *S. cerevisiae* | European | Wild |
| **YIIc17_E5** | *S. cerevisiae* | Mosaic, European | Fermentation |
| **YPS606** | *S. cerevisiae* | North American | Wild |
| **YPS128** | *S. cerevisiae* | North American | Wild |
| **YS9** | *S. cerevisiae* | Mosaic, European | Baking |
| **UWOPS03-461.4** | *S. cerevisiae* | Malaysia | Wild |
| **UWOPS05-217.3** | *S. cerevisiae* | Malaysia | Wild |
| **UWOPS05-227.2** | *S. cerevisiae* | Malaysia | Wild |
| **Y12** | *S. cerevisiae* | Sake | Fermentation |
| **NCYC110** | *S. cerevisiae* | Africa | Fermentation |
| **DBVPG6044** | *S. cerevisiae* | Africa | Fermentation |
| **DBVPG6765** | *S. cerevisiae* | European | Unknown |
| **L-1374** | *S. cerevisiae* | European | Fermentation |
| **L-1528** | *S. cerevisiae* | European | Fermentation |
| **UWOPS87-2421** | *S. cerevisiae* | Mosaic | Wild |
| **DBVPG1106** | *S. cerevisiae* | European | Fermentation |
| **UWOPS83-787.3** | *S. cerevisiae* | Mosaic | Wild |
| **BC187** | *S. cerevisiae* | European | Fermentation |
| **CBS432** | *S. paradoxus* | European | Wild |
| **N-17** | *S. paradoxus* | European | Wild |
| **CBS5829** | *S. paradoxus* | European | Wild |
| **T21.4** | *S. paradoxus* | European | Wild |
| **Y7** | *S. paradoxus* | European | Wild |
| **Y6.5** | *S. paradoxus* | European | Wild |
| **Q32.3** | *S. paradoxus* | European | Wild |
| **Q59.1** | *S. paradoxus* | European | Wild |
| **Q62.5** | *S. paradoxus* | European | Wild |
| **Q89.8** | *S. paradoxus* | European | Wild |
| **Q95.3** | *S. paradoxus* | European | Wild |
| **S36.7** | *S. paradoxus* | European | Wild |
| **Z1.1** | *S. paradoxus* | European | Wild |
| **Y9.6** | *S. paradoxus* | European | Wild |
| **Q74.4** | *S. paradoxus* | European | Wild |
| **Q96.8** | *S. paradoxus* | European | Wild |
| **LD7** | *S. paradoxus* | European | Wild |
| **Q31.4** | *S. paradoxus* | European | Wild |
| **Y8.5** | *S. paradoxus* | European | Wild |
| **Z1** | *S. paradoxus* | European | Wild |
| **Y8.1** | *S. paradoxus* | European | Wild |
| **KPN3829** | *S. paradoxus* | European | Wild |
| **YPS138** | *S. paradoxus* | American | Wild |
| **DBVPG6304** | *S. paradoxus* | American | Wild |
| **A12** | *S. paradoxus* | American | Wild |
| **N-44** | *S. paradoxus* | Far East | Wild |
| **IFO1804** | *S. paradoxus* | Far East | Wild |
