# Supplementary material for: Ancient Evolutionary Trade-Offs between Yeast Ploidy States
Source: PLoS Genet. 2013 Mar 21;9(3):e1003388. doi: 10.1371/journal.pgen.1003388 (PMC3605057; doi:10.1371/journal.pgen.1003388)
Supplement: Table S2 — Environments used in the screen. “Carbon source” indicates that 2% glucose was substituted with the indicated concentration of the relevant carbon source. “Nitrogen source” indicates that 0.5% ammonium sulfate was substituted with the relevant nitrogen sources at nitrogen limiting concentrations (corresponding to 29 µg N/mL). Except for the addition of 20 mg/L uracil, which cannot be used as nitrogen source by any of the strains, no other nitrogen was supplied. Furthermore, two consecutive pre-cultures were performed to deplete internal storages of nitrogen; the first was performed using nitrogen limiting amounts of ammonium sulfate, the second using nitrogen limiting amounts of the indicated nitrogen source. “Nutrient depletion” indicates that experiments were performed in medium completely lacking the indicated nutrient. (DOC) [file pgen.1003388.s008.doc]

**Table S2 Environments used in the screen**

**”Carbon source” indicates that 2% glucose was substituted with the indicated concentration of the relevant carbon source. “Nitrogen source” indicates that 0.5% ammonium sulfate was substituted with the relevant nitrogen sources at nitrogen limiting concentrations (corresponding to 29µg N/mL). Except for the addition of 20mg/L uracil, which cannot be used as nitrogen source by any of the strains , no other nitrogen was supplied. Furthermore, two consecutive pre-cultures were performed to deplete internal storages of nitrogen; the first was performed using nitrogen limiting amounts of ammonium sulfate, the second using nitrogen limiting amounts of the indicated nitrogen source. “Nutrient depletion” indicates that experiments were performed in medium completely lacking the indicated nutrient.**

| **Environment** | **Type** | **Classification** | **Concentration** |
| --- | --- | --- | --- |
| Cisplatin | DNA damage | DNA damage | 100 mg/mL |
| Doxorubicin | DNA damage | DNA damage | 20 µg/mL |
| Hydroxyurea | DNA damage | DNA damage | 15 mg/mL |
| Phleomycin | DNA damage | DNA damage | 5 µg/mL |
| Galactose | Nutrient limitation | Carbon source | 2 % |
| Nutrient excess, no stress (Glucose) | Nutrient limitation | Carbon source | 2 % |
| Glycerol | Nutrient limitation | Carbon source | 2 % |
| Maltose | Nutrient limitation | Carbon source | 8 % |
| Adenine | Nutrient limitation | Nitrogen source | 29 µg N/mL |
| Citrulline | Nutrient limitation | Nitrogen source | 29 µg N/mL |
| GABA | Nutrient limitation | Nitrogen source | 29 µg N/mL |
| Glycine | Nutrient limitation | Nitrogen source | 29 µg N/mL |
| Leucine | Nutrient limitation | Nitrogen source | 29 µg N/mL |
| Phenylalanine | Nutrient limitation | Nitrogen source | 29 µg N/mL |
| Threonine | Nutrient limitation | Nitrogen source | 29 µg N/mL |
| Tryptophan | Nutrient limitation | Nitrogen source | 29 µg N/mL |
| Urea | Nutrient limitation | Nitrogen source | 29 µg N/mL |
| (-) Biotin | Nutrient limitation | Nutrient depletion | No external biotin |
| (-) Inositol | Nutrient limitation | Nutrient depletion | No external inositol |
| (-) Magnesium | Nutrient limitation | Nutrient depletion | No external magnesium |
| (-) Pantothenate | Nutrient limitation | Nutrient depletion | No external pantothenate |
| (-) Zinc | Nutrient limitation | Nutrient depletion | No external zinc |
| CdCl_2_ | Toxin | Inorganic toxin | 150 µM |
| CuCl_2_ | Toxin | Inorganic toxin | 1 mM |
| LiCl | Toxin | Inorganic toxin | 225 mM |
| Sodium arsenite | Toxin | Inorganic toxin | 5 mM |
| NaCl | Toxin | Inorganic toxin | 1 M |
| Caffeine | Toxin | Organic toxin | 2.25 mg/mL |
| Cycloheximide | Toxin | Organic toxin | 0.025 µg/mL |
| Ethanol | Toxin | Organic toxin | 10 % |
| Paraquat | Toxin | Organic toxin | 400 µg/mL |
| Rapamycin | Toxin | Organic toxin | 1 µg/mL |
| Heat | Other | High temperature | 40 ˚C |

Warringer, J, E Zorgo, FA Cubillos, et al. 2011. Trait variation in yeast is defined by population history. PLoS Genet 7:e1002111.
