# Supplementary material for: Ancient Evolutionary Trade-Offs between Yeast Ploidy States
Source: PLoS Genet. 2013 Mar 21;9(3):e1003388. doi: 10.1371/journal.pgen.1003388 (PMC3605057; doi:10.1371/journal.pgen.1003388)
Supplement: Table S3 — Gene deletion strains used to screen for cell size effects on mitotic performance. Single gene deletions in the BY4741 (haploid) or, as homozygotes or heterozygotes, in the BY4743 (diploid) background used to screen for effects of cell size on mitotic performance in different environmental contexts (see Table S4). (DOC) [file pgen.1003388.s009.doc]

**Table S3 Gene deletion strains used to screen for cell size effects on mitotic performance**

Single gene deletions in the BY4741 (haploid) or, as homozygotes or heterozygotes, in the BY4743 (diploid) background used to screen for effects of cell size on mitotic performance in different environmental contexts (see Table S4).

| **ORF deleted** | **Gene deleted** | **Ploidy** | **Type of deletion** | **Cell size** |
| --- | --- | --- | --- | --- |
| ***YGL047W*** | *ALG13* | Diploid | Heterozygote | Large |
| ***YDR526C*** | *YDR526C* | Diploid | Heterozygote | Large |
| ***YDL007W*** | *RPT2* | Diploid | Heterozygote | Large |
| ***YPL129W*** | *TAF14* | Diploid | Homozygote | Large |
| ***YJL127C*** | *SPT10* | Diploid | Homozygote | Large |
| ***YCR077C*** | *PAT1* | Diploid | Homozygote | Large |
| ***YBR279W*** | *PAF1* | Diploid | Homozygote | Large |
| ***YDR264C*** | *AKR1* | Diploid | Homozygote | Large |
| ***YDR138W*** | *HPR1* | Diploid | Homozygote | Large |
| ***YJL115W*** | *ASF1* | Diploid | Homozygote | Large |
| ***YCR081W*** | *SRB8* | Diploid | Homozygote | Large |
| ***YNR052C*** | *POP2* | Diploid | Homozygote | Large |
| ***YLR131C*** | *ACE2* | Diploid | Homozygote | Large |
| ***YAL021C*** | *CCR4* | Diploid | Homozygote | Large |
| ***YAL040C*** | *CLN3* | Diploid | Homozygote | Large |
| ***YKL114C*** | *APN1* | Diploid | Homozygote | Large |
| ***YBR134W*** | *YBR134W* | Diploid | Homozygote | Large |
| ***YML014W*** | *TRM9* | Diploid | Homozygote | Large |
| ***YJR054W*** | *ERM6* | Diploid | Homozygote | Large |
| ***YDR335W*** | *MSN5* | Diploid | Homozygote | Large |
| ***YER167W*** | *BCK2* | Diploid | Homozygote | Large |
| ***YCR020W*** | *YCR020W* | Diploid | Homozygote | Large |
| ***YDR253C*** | *MET32* | Diploid | Homozygote | Large |
| ***YDL106C*** | *PHO2* | Diploid | Homozygote | Large |
| ***YHL009C*** | *YAP3* | Diploid | Homozygote | Large |
| ***YDL066W*** | *IDP1* | Diploid | Homozygote | Large |
| ***YNL139C*** | *THO2* | Diploid | Homozygote | Large |
| ***YGR056W*** | *RSC1* | Diploid | Homozygote | Large |
| ***YJL080C*** | *SCP160* | Diploid | Homozygote | Large |
| ***YLR131C*** | *ACE2* | Haploid MATa | Haploid | Large |
| ***YDL066W*** | *IDP1* | Haploid MATa | Haploid | Large |
| ***YDR264C*** | *AKR1* | Haploid MATa | Haploid | Large |
| ***YAL040C*** | *CLN3* | Haploid MATa | Haploid | Large |
| ***YJL127C*** | *SPT10* | Haploid MATa | Haploid | Large |
| ***YCR020W*** | *YCR020W* | Haploid MATa | Haploid | Large |
| ***YER167W*** | *BCK2* | Haploid MATa | Haploid | Large |
| ***YDR335W*** | *MSN5* | Haploid MATa | Haploid | Large |
| ***YGR056W*** | *Gene deleted* | Haploid MATa | Haploid | Large |
| ***YDR138W*** | *HPR1* | Haploid MATa | Haploid | Large |
| ***YDR224C*** | *HTB1* | Diploid | Heterozygote | Small |
| ***YGL097W*** | *SRM1* | Diploid | Heterozygote | Small |
| ***YPL254W*** | *HFI1* | Diploid | Homozygote | Small |
| ***YIL098C*** | *FMC1* | Diploid | Homozygote | Small |
| ***YKL212W*** | *SAC1* | Diploid | Homozygote | Small |
| ***YKL109W*** | *HAP4* | Diploid | Homozygote | Small |
| ***YGL237C*** | *HAP2* | Diploid | Homozygote | Small |
| ***YOR043W*** | *WHI2* | Diploid | Homozygote | Small |
| ***YJL095W*** | *BCK1* | Diploid | Homozygote | Small |
| ***YNL307C*** | *MCK1* | Diploid | Homozygote | Small |
| ***YIL052C*** | *RPL34B* | Diploid | Homozygote | Small |
| ***YLL016W*** | *SDC25* | Diploid | Homozygote | Small |
| ***YOR083W*** | *WHI5* | Diploid | Homozygote | Small |
| ***YOR008C*** | *SLG1* | Diploid | Homozygote | Small |
| ***YHR030C*** | *SLT2* | Diploid | Homozygote | Small |
| ***YMR304W*** | *UBP15* | Diploid | Homozygote | Small |
| ***YDR392W*** | *SPT3* | Diploid | Homozygote | Small |
| ***YAL056W*** | *GPB2* | Diploid | Homozygote | Small |
| ***YML051W*** | *GAL80* | Diploid | Homozygote | Small |
| ***YKL037W*** | *AIM26* | Diploid | Homozygote | Small |
| ***YGL237C*** | *HAP2* | Haploid *MATa* | Haploid | Small |
| ***YKL212W*** | *SAC1* | Haploid *MATa* | Haploid | Small |
| ***YKL109W*** | *HAP4* | Haploid *MATa* | Haploid | Small |
| ***YNL307C*** | *MCK1* | Haploid *MATa* | Haploid | Small |
| ***YLL016W*** | *SDC25* | Haploid *MATa* | Haploid | Small |
| ***YHR030C*** | *SLT2* | Haploid *MATa* | Haploid | Small |
| ***YPL254W*** | *HFI1* | Haploid *MATa* | Haploid | Small |
| ***YOR083W*** | *WHI5* | Haploid *MATa* | Haploid | Small |
| ***YKL037W*** | *AIM26* | Haploid *MATa* | Haploid | Small |
| ***YML051W*** | *GAL80* | Haploid *MATa* | Haploid | Small |
