# Supplementary material for: Ancient Evolutionary Trade-Offs between Yeast Ploidy States
Source: PLoS Genet. 2013 Mar 21;9(3):e1003388. doi: 10.1371/journal.pgen.1003388 (PMC3605057; doi:10.1371/journal.pgen.1003388)
Supplement: Table S4 — Environments tested in cell size and mating type locus experiments. (DOC) [file pgen.1003388.s010.doc]

**Table S4 Environments tested in cell size and mating type locus experiments**

| **Environment tested** | **Fitness component tested** |
| --- | --- |
| **Caffeine** | Lag |
| **Ethanol** | Lag |
| **Galactose** | Lag |
| **Maltose** | Lag |
| **Sodium arsenite** | Lag |
| **Heat** | Lag, Efficiency |
| **Paraquat** | Lag, Efficiency |
| **Doxorubicin** | Lag, Rate, Efficiency |
| **Rapamycin** | Lag, Rate, Efficiency |
| **Inositol (-)** | Rate |
| **Zinc (-)** | Rate |
| **CuCl_2_** | Rate, Efficiency |
| **Nutrient excess, no stress** | Rate, Efficiency |
| **Hydroxyurea** | Rate, Efficiency |
| **LiCl** | Rate, Efficiency |
